# The driving mechanism of psychological capital on employees’ innovative behavior: the mediating role of work engagement and the moderating role of coaching leadership

**DOI:** 10.3389/fpsyg.2026.1915517

**Published:** 2026-10-01

**Authors:** Rui Guo, Qing Liu, Yujie Wei

**Affiliations:** 1 Chongqing Metropolitan College of Science and Technology, Yongchuan, Chongqing, China; 2 Chongqing Industry and Information Vocational College, Chongqing, China; 3 Chongqing Wuyi Polytechnic, Chongqing, China

**Keywords:** coaching leadership, employees’ innovative behavior, knowledge workers, moderated mediation, psychological capital, work engagement

## Abstract

Employee innovative behavior is an important micro-level source of organizational competitiveness. Grounded in conservation of resources (COR) theory, with the job demands–resources (JD–R) model specifying the complementary motivational process, this study examines how PsyCap is associated with innovative behavior and whether this association varies with coaching leadership. Survey data from knowledge workers in Chinese enterprises showed a positive association between PsyCap and innovative behavior. Work engagement partially mediated this association. Coaching leadership was positively associated with the strength of the work engagement–innovative behavior relationship and of the indirect association between PsyCap and innovative behavior through engagement. The conditional indirect effect was supported at medium and high, but not low, levels of coaching leadership. These findings position work engagement as a motivational state through which employees invest sustained energy and attention, and coaching leadership as a contextual condition associated with the conversion of psychological resources into innovative action.

## 1 Introduction

Employee innovative behavior is a micro-foundation of organizational innovation because new products, processes, and routines often originate from employees’ generation, promotion, and implementation of novel ideas (Amabile, 1988; Anderson et al., 2014). Yet investments in incentives and digital transformation do not automatically produce sustained employee innovation. Whether such investments yield innovative action also depends on employees’ psychological resources and on a work context that enables those resources to be mobilized. Psychological capital (PsyCap)—a developable positive psychological state comprising self-efficacy, hope, optimism, and resilience—is therefore especially relevant: it has been associated with desirable employee attitudes and performance and, more specifically, with innovative workbehavior (Avey et al., 2011; Luthans and Youssef-Morgan, 2017; Chen et al., 2022; Kumar et al., 2022; Blasco-Giner et al., 2023).

The present study focuses on knowledge workers, defined as employees whose primary tasks involve the application of specialized expertise, complex problem-solving, and the generation of novel ideas or solutions (Drucker, 1959; Davenport, 2005). This population is particularly relevant for investigating the PsyCap—innovation relationship because their work demands sustained cognitive effort, creativity, and autonomous judgment—conditions under which psychological resources and developmental leadership are likely to be most consequential.

Despite this evidence, three related gaps remain. First, prior research has largely established direct or separate associations among PsyCap, work engagement, leadership, and innovative behavior, but has provided a less complete account of the sequential process by which a personal resource may be mobilized and associated with innovative action. Consequently, evidence that PsyCap is associated with innovation does not by itself explain the motivational mechanism connecting the two. Second, work engagement is frequently examined as an outcome of personal or job resources; its role as a proximal motivational state through which employees invest sustained energy and attention in the generation, promotion, and implementation of ideas remains insufficiently specified. Third, leadership research has commonly treated positive leadership styles as direct predictors of creativity or innovation. Less is known about whether a specifically developmental leadership context is associated with the extent to which engaged employees translate their vigor, dedication, and absorption into innovative behavior. These omissions leave both the process linking PsyCap with innovation and its contextual boundary incompletely understood.

To address these gaps, the present study develops a second-stage moderated mediation model grounded in COR theory. PsyCap is conceptualized as a valuable personal resource; work engagement captures a motivational state through which employees deploy energy, dedication, and attention; and innovative behavior is the behavioral correlate of that deployment. Coaching leadership is positioned as a contextual resource associated with how effectively engagement is translated into innovative behavior. The theoretical architecture is deliberately hierarchical rather than additive: COR theory supplies the overarching resource-acquisition, investment, and gain logic, whereas the JD–R model is used only as a complementary process framework to specify the motivational pathway from PsyCap to work engagement. Accordingly, the model links a mediator that helps explain how PsyCap is associated with innovative behavior with a moderator that indicates when the latter-stage association is stronger.

Recent systematic reviews have consolidated the evidence on psychological capital and innovative work behavior. Blasco-Giner et al. (2023) found that while PsyCap consistently predicts innovative work behavior as an antecedent, mediator, and moderator, the mechanisms through which this relationship operates remain insufficiently specified. The review particularly highlighted the need for more nuanced examinations of mediating pathways and contextual boundary conditions. Similarly, the meta-analysis by Avey et al. (2011) demonstrated that PsyCap is positively related to desirable employee attitudes, behaviors, and performance, yet called for greater attention to the processes linking PsyCap to specific behavioral outcomes.

Within the COR-based framework, the JD–R model serves a limited and complementary role by specifying the motivational process through which personal resources such as PsyCap foster work engagement and positive work outcomes (Bakker and Demerouti, 2024). Bakker and Demerouti also called for more attention to contextual boundary conditions that may strengthen or weaken these motivational pathways. In response, the present study introduces coaching leadership as a contextual resource that moderates the effectiveness of the PsyCap–engagement–innovation pathway.

Recent empirical studies have begun to unpack the mediating mechanisms linking psychological capital to innovative behavior. Hosseini et al. (2024) found that psychological capital mediates the effects of family, 65co-worker, and supervisor emotional support on innovative work behavior, highlighting the importance of affective factors in the innovation process. Subramani et al. (2024) demonstrated that specific components of psychological capital—particularly self-efficacy and optimism—mediate the relationship between transformational leadership and work engagement, which in turn influences innovative work behavior. These findings underscore the relevance of psychological capital as a transmission mechanism but also suggest that the leadership context in which employees operate may substantially affect how psychological resources are mobilized.

A particularly close comparison is Alwali (2024), who examined work engagement as a mediator between PsyCap and innovative work behavior and transformational leadership as a moderator of the direct PsyCap-innovation relationship among Iraqi nurses. The present study differs in both the leadership construct and the location of moderation. It examines coaching leadership—a development- and feedback-oriented context—as a moderator of the second-stage work engagement–innovation association rather than of the direct PsyCap–innovation association. The contribution is therefore incremental rather than a claim to be the first moderated-mediation study: it tests whether coaching leadership is associated with how effectively an existing state of engagement is translated into innovative behavior.

Coaching leadership has emerged as a promising contextual resource. Hwang et al. (2023) linked it to creative performance through psychological empowerment and constructive voice behavior, and other studies have associated coaching or managerial coaching with creative and innovative behavior (Zhu et al., 2020; Pajuoja et al., 2025; Zhang et al., 2021; Zhao, 2023; Zhao and Yang, 2023). Most of this research, however, has treated coaching leadership as a direct antecedent or the starting point of an indirect process. Less attention has been paid to its role as a boundary condition that helps employees convert existing engagement into innovation. From a COR perspective, coaching leadership may protect invested resources and improve the return on employees’vigor, dedication, and absorption.

The study makes three incremental contributions. First, it moves beyond documenting a PsyCap–innovation association by specifying and testing a sequence linking personal resource possession, a motivational state that supports sustained investment, and innovative action. Second, it repositions work engagement from a general desirable outcome to a proximal mechanism through which PsyCap may be associated with employees’ generation, promotion, and implementation of ideas. Third, it conceptualizes coaching leadership not simply as another direct antecedent of innovation, but as a second-stage boundary condition associated with how effectively engaged employees translate motivational energy into innovative behavior. By testing these roles simultaneously, the study examines both how and under what leadership conditions PsyCap is associated with employee innovation, thereby offering a more integrated account than studies of the relationships in isolation.

## 2 Theoretical background and hypotheses development

### 2.1 A COR-based theoretical framework

COR theory serves as the overarching theoretical foundation of this study. It explains why employees seek, protect, and invest resources and why an initial resource stock may generate further resource gains (Hobfoll, 1989). In the present model, PsyCap constitutes that initial personal-resource stock: self-efficacy supports confidence in solving innovation-related problems; hope sustains goal-directed effort when obstacles arise; optimism promotes constructive appraisal of uncertainty; and resilience enables recovery and renewed investment after setbacks. COR theory therefore connects all four focal constructs through one resource-conversion logic rather than supplying an explanation for only one isolated relationship. The JD–R model performs a narrower and non-competing role. It specifies the motivational process through which personal resources are associated with vigor, dedication, and absorption and, in turn, with positive work outcomes (Bakker and Demerouti, 2007, 2017, 2024). Thus, COR theory explainsthe overarching logic of resource investment and gain, whereas the JD–R model clarifies the proximal PsyCap–work engagement pathway. Work engagement consequently represents a motivational state that supports the deployment of energy and attention, not an additional resource category or an investment behavior in itself.

Coaching leadership is incorporated within the COR framework as a contextual resource. Its measured behaviors of guidance, facilitation, and inspiration provide a developmental context that may, in turn, foster empowerment and psychological safety. These measured behaviors and potential consequences can reduce the uncertainty and resource costs associated with proposing and implementing new ideas, allowing engaged employees to obtain greater behavioral value from the effort they invest. This logic supports its placement as a moderator of the work engagement–innovative behavior relationship. Field theory (Lewin, 1951) could provide a broad account of behavior as a function of the person and environment; however, it does not add a distinct mechanism needed to explain the focal second-stage interaction once coaching leadership is specified as a contextual resource within COR theory. Retaining it as a parallel foundation would therefore increase theoretical complexity without improving the model’s explanatory precision. For theoretical parsimony, it is not treated as a separate pillar. The resulting framework assigns a unique role to each retained perspective while maintaining one coherent resource-based explanation.

Innovation diffusion theory concerns the adoption and spread of innovations, whereas this study examines the motivational deployment of employees’ psychological resources; it is therefore outside the focal framework.

### 2.2 Psychological capital and employees’ innovative behavior

Psychological capital, formally defined by Luthans and Youssef (2004) as “an individual’s positive psychological state of development characterized by self-efficacy, hope, optimism, and resilience,” is a developable personal resource. Innovative behavior is resource intensive and uncertain: employees must devote attention to generating ideas, persist when ideas encounter resistance, and accept the possibility that implementation effort will not be rewarded. COR theory predicts that resource-rich individuals are more willing and able to invest resources in activities that may produce future gains, whereas employees with fewer resources are more likely to conserve what remains and avoid additional loss. PsyCap should therefore support innovation at each stage. Self-efficacy increases confidence in solving unfamiliar problems, 138 hope sustains the search for alternative pathways, optimism supports constructive appraisals of uncertain outcomes, and resilience enables employees to recover after rejection or failure. Together, these resources lower the perceived threat of resource loss and sustain effort toward the potential gains associated with successful innovation. Existing evidence linking PsyCap to innovative behavior is consistent with this resource-based reasoning (Avey et al., 2011; Newman et al., 2014; Chen et al., 2022; Kumar et al., 2022; Wang and Wang, 2023; Wang and Zhu, 2024; Zheng and Ni, 2022).

PsyCap fits the COR-based question because, unlike relatively stable dispositions such as proactive personality or locus of control, it is a developable, state-like resource (Luthans et al., 2007; Luthans and Youssef-Morgan, 2017). Its four capacities also correspond to the confidence, persistence, constructive appraisal, and recovery required across innovation stages. We therefore expect employees with greater PsyCap to exhibit more innovative behavior.

*H1*: Psychological capital is positively related to employee innovative behavior.

### 2.3 The mediating role of work engagement

Work engagement is a positive, fulfilling work-related state characterized by vigor, dedication, and absorption (Schaufeli et al., 2002). COR theory suggests that individuals who possess a stronger resource stock are better positioned to invest energy without becoming overly concerned about depletion. Employees with high PsyCap approach difficult goals with confidence, identify alternative routes when obstacles arise, expect that effort can lead to valued outcomes, and recover from setbacks. These resources reduce the psychological cost of sustained work investment. Consistent with the JD-R motivational process, PsyCap should therefore foster vigor, dedication, and absorption (Bakker and Demerouti, 2007, 2017, 2024). Accordingly:

*H2a*: Psychological capital is positively related to work engagement.

Work engagement should, in turn, facilitate innovative behavior because innovation requires more than having a potentially useful idea. Vigor supplies the energy needed to explore and test alternatives; dedi-cation gives innovation-related effort personal significance; and absorption sustains attention during idea development and implementation. These forms of motivational investment help employees persist through uncertainty, obtain support for their ideas, and overcome implementation barriers. Thus, engagement provi-des the behavioral energy through which employees pursue the resource gains associated with successful innovation (Scott and Bruce, 1994; Kwon and Kim, 2020; Huang et al., 2024). Accordingly:

*H2b*: Work engagement is positively related to employee innovative behavior.

Creative self-efficacy and creative-process engagement are plausible task-proximal alternatives, but the present study concerns sustained motivation across idea generation, promotion, and implementation. Work engagement is therefore selected because vigor, dedication, and absorption correspond to the broader COR-based investment process across the full innovative-behavior sequence. Future research should compare these mechanisms directly. Combining the two paths yields the proposed mediation mechanism. PsyCap provides personal resources that make sustained motivational investment more likely; engagement represents a positive motivational state through which employees apply vigor, dedication, and absorption; and innovative behavior is associated with that sustained application of energy and attention. PsyCap may still be associated with innovation through other routes, but COR theory and the JD-R motivational process jointly imply that part of its association with innovative behavior should operate through work engagement. Accordingly:

*H2c*: Work engagement mediates the relationship between psychological capital and employee innovative behavior.

### 2.4 The moderating role of coaching leadership

Coaching leadership refers to a developmental style in which leaders guide, facilitate, and inspire subordinates to recognize their potential, overcome challenges, and improve performance (Heslin et al., 2006; Zhu et al., 2020; Li and Hsu, 2022). Unlike command-and-control leadership, it is developmental, enabling, and heuristic. COR theory implies that a contextual resource is most consequential when it protects employees’invested resources or increases the likelihood that their investment produces gains. Engagement gives employees energy and persistence, but converting that motivation into innovation can still be costly: ideas may be ambiguous, require managerial support, or expose employees to criticism and failure. Guidance, facilitation, and inspiration can clarify goals and provide developmental feedback and access to resources; these behaviors may subsequently foster empowerment and psychological safety. The distinction is important: empowerment and psychological safety are theorized consequences of coaching behaviors in the present account, not dimensions measured by the coaching-leadership scale. Together, these processes may reduce the potential costs of innovation and increase the expected return on the vigor, dedication, and absorption employees invest (Hwang et al., 2023; Pajuoja et al., 2025).

Other positive leadership styles may promote innovation through vision, values, or socioemotional support. Coaching leadership is especially relevant to the focal second stage because its guidance, facilitation, and inspiration may help engaged employees advance and implement ideas and may foster empowering and psychologically safe conditions. It is examined as a context-specific resource, not as a universally superior leadership style.

*H3*: Coaching leadership positively moderates the relationship between work engagement and employees’ innovative behavior, such that the relationship is stronger when coaching leadership is high.

The same reasoning produces a conditional indirect effect. The first stage of the proposed process concerns whether PsyCap supplies resources that support engagement; the second concerns whether that invested motivation is converted into innovative behavior. Because coaching leadership strengthens the second stage, it should also change the magnitude of the overall indirect association. When coaching leadership is high, guidance, facilitation, and inspiration provide developmental support and may foster empowerment and psychological safety, making it more likely that engagement generated from PsyCap is associated with innovative action. When coaching leadership is low, engaged employees receive less assistance in overcoming the uncertainty and implementation costs of innovation, so the indirect association should be weaker. Accordingly:

*H4*: Coaching leadership moderates the mediating role of work engagement in the relationship between psychological capital and innovative behavior, such that the indirect effect is stronger when coaching leadership is high.

Figure 1 presents the conceptual framework.

**Figure 1:**
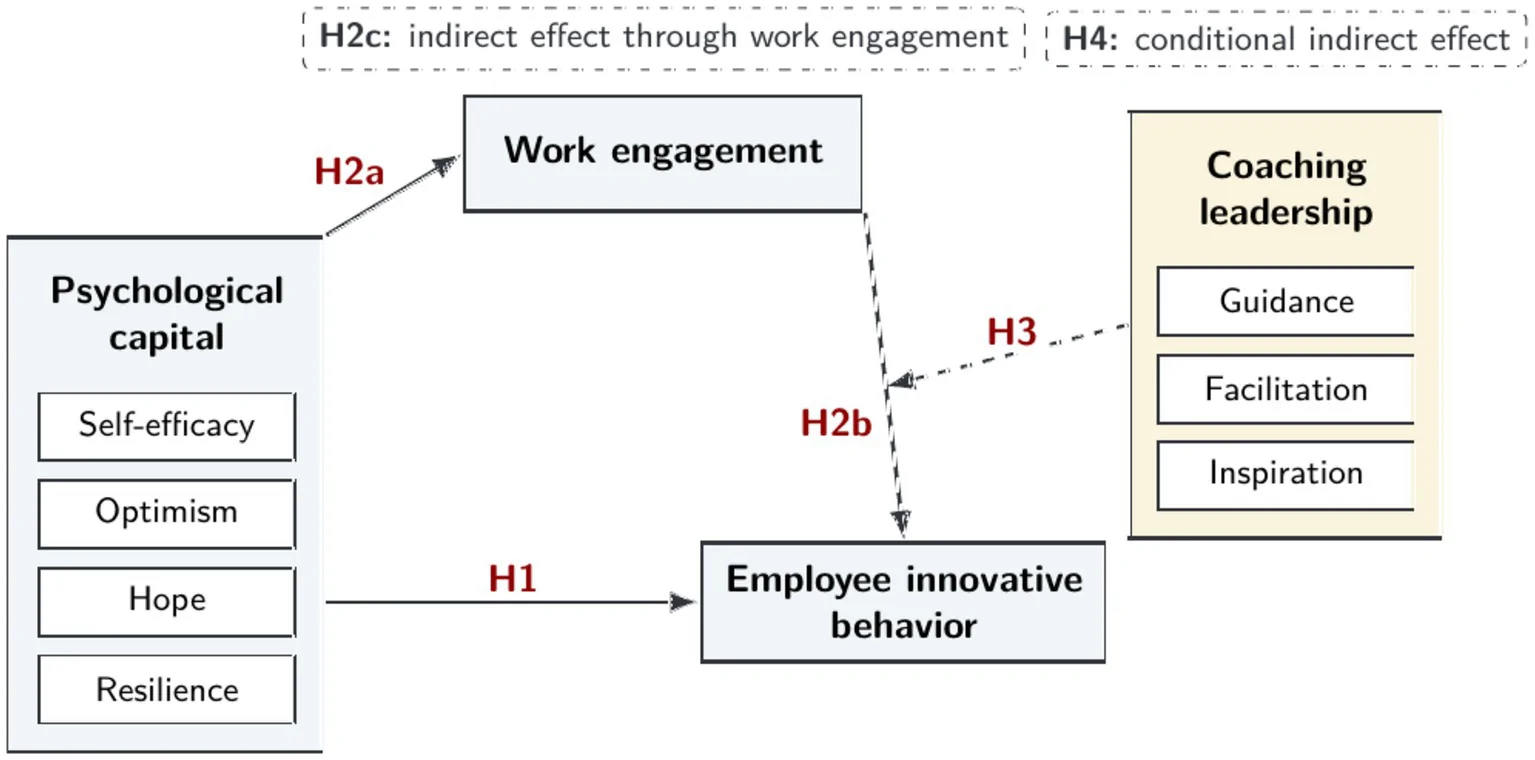
Conceptual framework of the moderated mediation model. H2c denotes the indirect effect of psychological capital on employee innovative behavior through work engagement. H3 denotes moderation of the second-stage path, and H4 denotes the corresponding conditional indirect effect.

## 3 Research design and methodology

### 3.1 Participants and procedure

This study employed a cross-sectional survey design targeting knowledge workers in knowledge-intensive organizations. A purposive convenience sampling strategy was adopted. Organizations with a relatively high concentration of knowledge workers were prioritized for recruitment. Access to participating organizations was obtained through organizational contacts and human resource departments, and questionnaires were distributed to eligible employees with the assistance of human resource managers or designated organizational representatives. Both online and paper-based questionnaires were used.

All questionnaires were completed anonymously. No personally identifiable information, such as names, telephone numbers, or organizational identifiers, was collected. Participants were informed that the survey was conducted solely for academic research purposes, that all responses would remain confidential, and that there were no right or wrong answers. To enhance data quality, the survey platform restricted duplicate submissions, and invalid or inconsistent responses were identified through attention checks, logical consistency checks, and subsequent manual screening. These procedures were implemented to reduce evaluation apprehension, social desirability bias, and common method bias.

The study was approved by the Ethics Committee of Chongqing University (Approval no. 2025-KLS-046) and conducted in accordance with applicable institutional and legal requirements. Written informed consent was obtained from all participants prior to participation.

A total of 600 questionnaires were distributed, and 452 responses were returned, yielding a response rate of 75.3%. After excluding 42 invalid questionnaires due to excessively short completion times or identical responses across all items, 410 valid questionnaires were retained for analysis. The valid responses accounted for 90.7% of the returned questionnaires and 68.3% of the total questionnaires distributed.

The final sample covered multiple provincial-level regions in China, with respondents concentrated in Chongqing, Beijing, Shanghai, Guangdong, and Zhejiang. The detailed demographic characteristics of the sample are presented in Table 1. Accordingly, the population to which the findings most directly pertain can be characterized as knowledge workers employed in participating knowledge-intensive organizations across multiple regions of China. In terms of industry distribution, the sample was primarily drawn from human resources services (52.0%), education and training (21.0%), and corporate consulting (12.4%), with the remaining industries—smart manufacturing (6.8%), high-tech (5.6%), and fintech (2.2%). Of the respondents, 42.0% were male and 58.0% were female; 54.1% were aged between 26 and 35 years, and 27.1% were aged between 36 and 45 years. Regarding educational attainment, 71.7% held a bachelor’s degree, 17.3% held a master’s degree or above, and 11.0% had a junior college education or below. Regarding functional roles, 34.8% were consultants, analysts, or project managers; 26.4% worked in HR, audit, IT, or finance; 21.2% in marketing, brand, or sales; and 17.6% in R&D, design, production, or operations. Regarding organizational position, 50.0% were frontline employees, 30.0% were middle-level managers, and 20.0% were senior managers.

**Table 1:** Demographic characteristics of respondents (*N* = 410).

Variable	Category	Frequency	Percentage (%)
Gender	Male	172	42.0
	Female	238	58.0
Age	26–35 years	222	54.1
	36–45 years	111	27.1
	Other age groups	77	18.8
Education level	Associate degree or below	45	11.0
	Bachelor’s degree	294	71.7
	Master’s degree and above	71	17.3
Region	Chongqing	184	45.0
	Shanghai	42	10.2
	Beijing	22	5.4
	Guangdong	18	4.4
	Zhejiang	15	3.6
	Other provinces	129	31.4
Industry	High-tech	23	5.6
	Fintech	9	2.2
	Education/Training	86	21.0
	Smart manufacturing	28	6.8
	Corporate consulting	51	12.4
	Human resources services	213	52.0
Department/function	R&D/Design/Production/Operations	72	17.6
	Marketing/Brand/Sales	87	21.2
	HR/Audit/IT/Finance	108	26.4
	Consultant/Analyst/Project Manager	143	34.8
Job classification	Frontline employee	205	50.0
	Middle management personnel	123	30.0
	Senior management personnel	82	20.0

Respondents were recruited using purposive convenience sampling from knowledge-intensive organizations. Although valid responses were obtained from 25 provincial-level regions in China, the sample was concentrated in Chongqing, Beijing, Shanghai, Guangdong, and Zhejiang. This regional distribution reflects both the concentration of knowledge-intensive organizations and the practical availability of organizational access rather than a nationally representative sampling design. Therefore, the findings should be interpreted as applying primarily to knowledge workers employed in participating knowledge-intensive organizations across multiple regions of China rather than to the Chinese workforce as a whole.

### 3.2 Measures

All scales used a five-point Likert response format (1 = strongly disagree, 5 = strongly agree). Psych-ological capital was measured with a 10-item shortened scale based on Luthans et al. (2007), covering self-efficacy, hope, optimism, and resilience (*α* = 0.883). Work engagement was measured with the nine-item short Utrecht Work Engagement Scale (Schaufeli et al., 2006; *α* = 0.934). Coaching leadership was measured with a 10-item scale covering guidance, inspiration, and empowerment (*α* = 0.907). Employees’innovative behavior was measured with an eight-item scale (Scott and Bruce, 1994; *α* = 0.922). Age, education, tenure, job position, and technical title were included as controls.

### 3.3 Data analysis strategy

Data were analyzed using SPSS 25.0 and AMOS 24.0. First, descriptive statistics, reliability coefficients, and correlations were calculated. Second, confirmatory factor analysis (CFA) evaluated measurement-model fit and discriminant validity. Common method variance was assessed using Harman’s single-factor diagnostic, an unmeasured latent method-factor comparison, and a full-collinearity VIF assessment. Third, hierarchical regression tested main, mediation, and moderation effects; variables involved in interactions were mean-centered. Fourth, mediation and moderated mediation were tested using ordinary least squares conditional-process models and 5,000 nonparametric bootstrap resamples with 95% percentile confidence intervals. The moderated-mediation specification corresponded to PROCESS Model 14 because coaching leadership was theorized to moderate the second stage of the mediation path.

## 4 Data analysis and results

### 4.1 Common method bias test

Because all focal variables came from the same respondents at one time point, we used three statistical diagnostics. First, the unrotated Harman analysis extracted nine factors with eigenvalues greater than 1; the first explained 38.978% of the total variance, indicating no dominant single-factor structure. Second, following Podsakoff et al. (2003), a common method factor was added to the hypothesized four-factor model. Consistent with the substantive CFA, this comparison used 24 observed indicators: four PsyCap dimension scores, nine work-engagement items, three coaching-leadership dimension scores, and eight employee-innovative-behavior items. The method-factor model fitted well (*χ*^2^ = 358.785, df = 222, *χ*^2^/df = 1.616, CFI = 0.977, TLI = 0.972, and RMSEA = 0.039). Although its chi-square was lower than that of the substantive model (∆*χ*^2^ = 80.003, ∆df = 24, *p* < 0.001), the approximate fit changes were small (∆CFI = 0.009, ∆TLI = 0.008, and ∆RMSEA = 0.005). Third, a full-collinearity assessment (Kock, 2015) produced construct VIFs of 1.577–2.018, all below 3.3. Together, the diagnostics suggest that common method variance did not dominate the observed construct structure, but they cannot establish that common method bias is absent.

### 4.2 Confirmatory factor analysis

A confirmatory factor analysis was conducted to evaluate whether psychological capital, work engage-ment, coaching leadership, and employee innovative behavior represented empirically distinct constructs. The measurement model contained 24 observed indicators. PsyCap was represented by four theoretically defined dimension scores (self-efficacy, hope, optimism, and resilience), coaching leadership by three dimension scores, work engagement by its nine items, and employee innovative behavior by its eight items. We compared the hypothesized four-factor model with three alternative models. In the three-factor model, psychological capital and work engagement were combined; in the two-factor model, psychological capital, work engagement, and coaching leadership were combined; and in the single-factor model, all 24 indicators included in the specified measurement model were loaded onto one factor. Model fit was evaluated using the *χ*^2^/df ratio, CFI, TLI, RMSEA, and SRMR. Values of *χ*^2^/df below 3, CFI and TLI above 0.90, and RMSEA and SRMR below 0.08 were considered indicative of acceptable fit.

As shown in Table 2, the hypothesized four-factor model demonstrated a good fit to the data (*χ*^2^/df = 1.784, CFI = 0.968, TLI = 0.964, RMSEA = 0.044, and SRMR = 0.035). Although the three-factor model also showed an acceptable fit, the four-factor model fitted the data significantly better (∆*χ*^2^ = 196.627, ∆df = 3, *p* < 0.001). The two-factor and single-factor models produced substantially poorer fit indices.

**Table 2:** Confirmatory factor analysis.

Model	*χ*^2^/df	CFI	TLI	RMSEA	SRMR
Four - factor model	1.784	0.968	0.964	0.044	0.035
Three - factor model	2.552	0.936	0.929	0.062	0.061
Two - factor model	3.368	0.901	0.892	0.076	0.071
Single -factor model	7.101	0.745	0.721	0.122	0.090

The 24 indicators comprised four PsyCap dimension scores, nine work-engagement items, three coaching-leadership dimension scores, and eight employee-innovative-behavior items. In the hypothesized four-factor model, the four constructs were modeled as distinct. In the three-factor model, psychological capital and work engagement were combined. In the two-factor model, psychological capital, work engagement, and coaching leadership were combined. In the single-factor model, all 24 indicators were loaded onto one factor.

Taken together, these results support the discriminant validity of the four focal constructs.

All standardized factor loadings in the hypothesized four-factor model exceeded 0.50 (Table 3). Com-posite reliability (CR) ranged from 0.753 to 0.934 and therefore exceeded 0.70 for every construct. The average variance extracted (AVE) exceeded 0.50 for work engagement, coaching leadership, and employee. The AVE for psychological capital was 0.435. Although this value is below the conventional 0.50 guideline, the construct retained acceptable composite reliability and all four standar- dized loadings exceeded 0.50. As a published precedent, treated constructs with loadings above 0.50, CR above 0.70, and AVE above 0.40 as reliable and unidimensional. On this basis, the convergent measurement quality of psychological capital was considered acceptable but comparatively weaker and is interpreted cautiously.

**Table 3:** Standardized factor loadings, composite reliability, and average variance extracted.

Construct	Indicators	Loading range	CR	AVE
Psychological capital	4 dimension scores	0.581–0.754	0.753	0.435
Work engagement	9 items	0.633–0.882	0.934	0.615
Coaching leadership	3 dimension scores	0.717–0.790	0.786	0.551
Employee innovative behavior	8 items	0.674–0.851	0.923	0.602

CR, Composite reliability; AVE, Average variance extracted. CR and AVE were calculated from the standardized loadings reported for the hypothesized four-factor measurement model.

### 4.3 Reliability analysis

All Cronbach’s alpha coefficients exceeded 0.70 (Table 4), indicating good internal consistency.

**Table 4:** Reliability test results.

Variable	Cronbach’s α
Psychological capital	0.883
Work engagement	0.934
Coaching leadership	0.907
Employees’ innovative behavior	0.922

### 4.4 Descriptive statistics and correlations

Hierarchical regression results are summarized in Table 5. The seven models retain the original analyticalsequence, with corrected dependent-variable headings, coefficient labels, and incremental R2 comparisons.

**Table 5:** Means, standard deviations, correlations, and VIF.

Variables	1	2	3	4	5	6	7	8	9	M	SD
Age	–									2.193	0.730
Education	0.028	–								2.056	0.550
Work tenure	0.679**	−0.147**	–							3.690	1.359
Job position	0.363**	0.036	0.378**	–						1.688	0.772
Technical title	0.274**	0.211**	0.183**	0.325**	–					1.732	0.931
Psychological capital	0.039	0.073	0.094	0.145**	0.111*	(1.993)				3.970	0.667
Work engagement	0.045	−0.045	0.166**	0.196**	0.138**	0.618**	(1.969)			3.921	0.788
Coaching leadership	−0.054	−0.056	0.198**	0.083	0.091	0.439**	0.541**	(1.577)		3.734	0.791
Innovative behavior	0.072	0.096	0.131**	0.250**	0.149**	0.636**	0.580**	0.534**	(2.018)	3.963	0.699

*N* = 410. Entries are unstandardized regression coefficients (B); standard errors are in parentheses. For Model 2, ∆R2 is relative to Model 1; for Model 3, it is relative to the controls-only Model 5; for Models 4, 6, and 7, it is relative to Models 3, 5, and 6, respectively. *p < 0.05; **p < 0.01; ***p < 0.001 (two-tailed).

#### 4.4.1 Main association (H1)

With innovative behavior as the dependent variable, the control variables explained 8.2% of the variance. Adding PsyCap increased the explained variance to 43.4% (∆R2 = 0.352). PsyCap had a positive unstandardized regression coefficient (B = 0.633, *p* < 0.001), supporting H1.

#### 4.4.2 Mediation effect (H2a, H2b, H2c)

PsyCap was positively associated with work engagement (*B* = 0.705, *p* < 0.001), supporting H2a. When PsyCap and work engagement were entered together, engagement had a positive unstandardized coefficient for innovative behavior (*B* = 0.256, *p* < 0.001), supporting H2b. The coefficient for PsyCap remained positive but decreased to 0.453 (*p* < 0.001). The bootstrapped indirect effect through engagement was 0.181, with a 95% confidence interval of [0.097, 0.271]. Because the interval excluded zero, the results supported partial mediation and H2c (Table 6).

**Table 6:** Bootstrap results for mediation.

Effect type	Effect	SE	95% CI lower	95% CI upper
Indirect effect through work engagement	0.181	0.044	0.097	0.271
Direct effect	0.453	0.048	0.358	0.548

#### 4.4.3 Moderation effect

The interaction between work engagement and coaching leadership was significant (*B* = 0.174, *p* < 319 0.001), supporting H3. The association between work engagement and innovative behavior was stronger under high coaching leadership [effect = 0.512, 95% CI (0.412, 0.612)] than under low coaching leadership [effect = 0.236, 95% CI (0.151, 0.322)] (Table 7).

**Table 7:** Bootstrap results for mediation.

Effect type	Effect	SE	95% CI lower	95% CI upper
Indirect effect through work engagement	0.181	0.044	0.097	0.271
Direct effect	0.453	0.048	0.358	0.548

#### 4.4.4 Moderated mediation

An ordinary least squares conditional-process model corresponding to PROCESS Model 14 was used to test conditional indirect effects. Re-estimation from the final item-level dataset with 5,000 nonparametric bootstrap resamples yielded an index of moderated mediation of 0.124, 95% percentile CI [0.062, 0.183], excluding zero. The conditional indirect effect of psychological capital on innovative behavior through work engagement was significant at medium [effect = 0.136, 95% CI (0.063, 0.223)] and high levels of coaching leadership [effect = 0.234, 95% CI (0.150, 0.331)], but not significant at low coaching leadership [effect = 0.038, 95% CI (−0.047, 0.141)]. Thus, H4 was supported (Table 8).

**Table 8:** Conditional indirect effects of psychological capital on innovative behavior through work engagement.

Coaching leadership	Effect	SE	95% CI lower	95% CI upper
Low (M-SD)	0.038	0.048	−0.047	0.141
Medium (M)	0.136	0.041	0.063	0.223
High (M + SD)	0.234	0.046	0.150	0.331
High–low difference	0.196	0.048	0.098	0.289
Index of moderated mediation	0.124	0.030	0.062	0.183

## 5 Discussion

### 5.1 Interpretation of findings

The positive PsyCap–innovation association is consistent with broader PsyCap evidence (Avey et al., 2011; Newman et al., 2014) and innovation-specific studies (Chen et al., 2022; Blasco-Giner et al., 2023). Innovation involves uncertain returns, resistance, and possible failure. Self-efficacy and hope support problehm solving and alternative pathways, whereas optimism and resilience help employees continue after rejection or implementation setbacks. The four resources therefore correspond to demands arising at different innovation stages.

The partial mediation result extends evidence on motivational and cognitive mechanisms linking PsyCap with innovation (Chen et al., 2022; Kumar et al., 2022; Blasco-Giner et al., 2023) and on engagement as an innovation correlate (Kwon and Kim, 2020). Vigor, dedication, and absorption may sustain demanding innovation activities, but the remaining direct association leaves room for creative confidence, proactive coping, and persistence after idea rejection. Engagement is therefore important but not exhaustive as an explanatory pathway.

The moderation finding complements studies linking coaching with creativity and innovative behavior (Hwang et al., 2023; Pajuoja et al., 2025) but suggests a more specific boundary role. Engaged employees may still lack implementation clarity, psychological safety, or managerial support. The measured coaching behaviors of guidance, facilitation, and inspiration may reduce these barriersdirectly and may also foster empowerment and psychological safety, which is consistent with the stronger engagement-innovation association under higher coaching leadership. The conditional indirect pattern is compatible with evidence that leadership context can qualify PsyCap-related innovation processes (Blasco-Giner et al., 2023) and extends this reasoning from participative to coaching leadership. The nonsignificant estimate at low coaching leadership does not show that PsyCap or engagement is valueless; it indicates that this sample provided no clear evidence of the indirect association when developmental guidance was limited.

### 5.2 Theoretical implications

First, the study extends the application of COR theory by distinguishing personal-resource possession (PsyCap), a motivational state that supports sustained investment of energy and attention (work engagement), and behavior associated with that investment (innovation). Coaching leadership adds boundary precision by indicating that the engagement-innovation association varies across developmental leadership contexts.

This staged account applies COR reasoning to discretionary and uncertain behavior without claiming to modify the theory’s core propositions. Second, the study connects PsyCap and engagement research. PsyCap predicts desirable attitudes and behaviors (Avey et al., 2011; Newman et al., 2014), while personal and job resources commonly precede engagement (Bakker and Demerouti, 2017, 2024). Positioning engagement between PsyCap and innovation treats it as a proximal motivational mechanism rather than only a favorable endpoint; partial mediation also shows that other mechanisms remain plausible. Third, the study shifts coaching-leadership research from a purely direct-effect account toward a boundary- condition account. Guidance, facilitation, and inspiration are the coaching behaviors represented in the measure; these behaviors may provide developmental feedback and goal clarification and may foster empowerment and psychological safety, thereby helping engaged employees manage the uncertainty and potential loss involved in advocating and implementing ideas.

Fourth, the model integrates a personal antecedent, a motivational mechanism, and a proximal leadership condition. Compared with studies using a different leadership boundary, such as participative leadership (Blasco-Giner et al., 2023), it identifies coaching leadership as a developmental context that may help explain variation in innovative behavior among similarly engaged employees.

### 5.3 Practical implications

The results suggest a coordinated set of actions for human-resource units, line managers, and leadership-development teams. Because the design is observational, these recommendations should be introduced as pilot interventions and evaluated rather than treated as guaranteed causal solutions.

First, human-resource units can pilot a structured PsyCap-development program instead of relying on general motivational training. A short program could combine mastery-based assignments and peer modeling for self-efficacy, goal and pathway exercises for hope, evidence-based reframing for optimism, and setback-review and recovery planning for resilience. Organizations can assess PsyCap, engagement, and idea submission or implementation before the program, immediately afterward, and at a later follow-up. Comparing participating units with similar non-participating units would help managers determine whether the program is useful in their own setting before scaling it.

Second, line managers can create conditions that allow psychological resources to be invested through engagement. Practical actions include quarterly job-resource reviews, clear but challenging innovation goals, discretion over how tasks are completed, timely developmental feedback, and access to the information and cross-functional contacts needed to advance an idea. Managers can use brief pulse surveys and one-to-one conversations to monitor vigor, dedication, workload, and obstacles to implementation. Engagement should not be pursued by simply increasing effort; workloads and recovery opportunities should be reviewed so that high involvement does not become exhaustion.

Third, leadership-development teams can translate coaching leadership into observable routines. Training can require managers to practice goal clarification, open-ended questioning, active listening, developmental feedback, and delegation using innovation-related scenarios. These skills can be reinforced through monthly coaching conversations, peer observation, and 360-degree feedback. Evaluation should focus on behaviors—such as the quality and timeliness of feedback, employee voice, and follow-through on ideas—rather than attendance at training alone. The conditional pattern in this study suggests that PsyCap and engagement initiatives are more likely to be useful when managers also provide an environment in which employees can safely propose and implement ideas.

Finally, organizations can integrate these actions in a staged pilot: develop employee PsyCap, redesign selected job resources, train the responsible managers, and track both proximal indicators (PsyCap, engagement, psychological safety, and idea submissions) and distal indicators (ideas implemented and process improvements). Intervention content should be adapted to organizational size, industry, and local norms, and effectiveness should be reassessed before wider rollout. This coordinated approach is more consistent with the findings than implementing isolated motivational workshops or leadership training without changes to employees’work context.

### 5.4 Limitations and future directions

While this study makes meaningful theoretical and practical contributions, several limitations should be acknowledged, and these limitations point to promising directions for future research.

First, the cross-sectional design limits causal inference and cannot establish temporal order. Reverse causality is plausible: successful innovative behavior may strengthen PsyCap through mastery and positive feedback, while engagement and innovation may reinforce one another over time. Longitudinal cross-lagged designs could compare these competing directions, and diary or experience-sampling studies could examine shorter-term reciprocal dynamics. Field experiments or carefully designed quasi-experiments would provide stronger evidence about the effects of PsyCap development or coaching interventions.

Second, the observational estimates may be affected by endogeneity. Omitted variables—including proactive personality, job autonomy, innovation climate, leader–member exchange, and organizational support—could influence both the proposed predictors and innovative behavior. Non-random selection into teams with coaching-oriented leaders may also create selection bias. The control variables and theory-consistent associations reduce neither possibility to zero. Future research should collect relevant alternative predictors, use multiwave or panel data with unit or individual fixed effects where feasible, and apply designs such as matched comparisons, natural experiments, or valid instrumental-variable approaches when their assumptions can be justified. Sensitivity analyses should also be reported to show how robust the conclusions are to plausible unobserved confounding.

Third, the reliance on single-source, self-report measures introduces the possibility of common method variance and social-desirability bias. Although the procedural remedies, latent method-factor comparison, and full-collinearity assessment suggested that common method variance did not dominate the observed relationships, these diagnostics cannot establish that such bias is absent. Future research should use temporally separated measurement waves and incorporate supervisor ratings, peer evaluations, objective innovation indicators, and archival or behavioral data.

Fourth, the convenience sample limits population and cross-cultural generalizability. Participants were recruited through accessible Chinese enterprises and organizational contacts rather than probability sam-pling, so employees or firms willing to participate may differ systematically from those not reached. The concentration in five economically developed provincial-level regions, together with the relatively high education and tenure of the sample, further limits representation of Chinese employees as a whole. In addition, collectivism, power distance, harmony orientation, and institutional conditions may shape how employees interpret coaching and innovative behavior. The findings should therefore not be generalized automatically to other Chinese regions, industries, occupational groups, or national cultures. Replication should use stratified or probability-based samples where feasible, include a broader range of firms and regions, and test measurement invariance and structural paths across countries.

Fifth, the study’s exclusive focus on knowledge workers may limit the generalizability of the findings to other occupational groups, such as manual workers, service workers, or routine-task employees whose work involves less cognitive complexity and discretionary innovation. The mechanisms proposed here—particularly the mobilization of psychological resources through work engagement and the moderating role of coaching leadership—may operate differently when tasks are more standardized or when innovation is not an expected role requirement. Future research should examine whether the present moderated mediation model replicates across diverse occupational categories and task environments.

Finally, the model does not exhaust the relevant resources, mechanisms, or consequences. Future research should compare coaching leadership with other leadership and organizational conditions (Zhao and Mohd Kamil, 2025), examine the distinct roles of PsyCap components (Subramani et al., 2024), and test alternative mechanisms such as creative self-efficacy, knowledge sharing, autonomous motivation, and psychological empowerment. Potential nonlinear or adverse outcomes—including workaholism, burnout, or coaching perceived as excessive interference—also warrant study. Such extensions could clarify both when the proposed moderated mediation pattern generalizes and where its limits lie.

## 6 Conclusion

This study supports a contingent resource-conversion account of innovative behavior: PsyCap represents a developable personal-resource stock, work engagement is a motivational state through which knowledge workers apply sustained energy and attention, and coaching leadership is associated with how effectively engagement is translated into innovative action. The principal theoretical advance is to distinguish resource possession, motivational activation, and context-dependent conversion within one COR-based model rather than treating PsyCap, engagement, and leadership as independent predictors. Without claiming that these resources operate identically across all occupational groups.

For practice, coordinated PsyCap development, engagement-supporting job design, and coaching are more likely to support innovation than isolated initiatives. The model also directs future research toward longitudinal, multisource, experimental, and cross-cultural tests before strong causal or universal claims are made.

## Data Availability

The raw data supporting the conclusions of this article will be made available by the authors, without undue reservation.
